# A Candidate Salivary miRNA Panel for Bronchopulmonary Dysplasia in Very and Extremely Low-Birth-Weight Preterm Infants: A Pilot Exploratory Study

**DOI:** 10.3390/life16071202

**Published:** 2026-07-21

**Authors:** Arailym Abilbayeva, Elmira Bitanova, Iskander Isgandarov, Beibitgul Bizhigitova, Dinara Yelyubayeva, Kristina Kovaleva, Zhanar Akhmetova, Ismira Gassanova, Balaussa Seitkhan, Indira Baibolsynova, Aibek Smagul, Zhuldyz Zhoshiyeva, Nishankul Bozhbanbayeva

**Affiliations:** 1 Shortanbayev General Immunology Department, Asfendiyarov Kazakh National Medical University, Almaty 050000, Kazakhstan; bitanova.e@kaznmu.kz (E.B.); bijigitova.b@kaznmu.kz (B.B.); 2 Laboratory of Breeding and Biotechnology, Institute of Plant Biology and Biotechnology, Almaty 050000, Kazakhstan; alexelite555@gmail.com; 3 Department of Highly Dependent Newborns, Center for Perinatology and Pediatric Cardiac Surgery, Almaty 050000, Kazakhstan; dina__1992@mail.ru; 4 Genome Centre, Asfendiyarov Kazakh National Medical University, Almaty 050000, Kazakhstan; kovaleva.k@kaznmu.kz (K.K.); akhmetovazhn@gmail.com (Z.A.); 5 School of Pediatrics, Asfendiyarov Kazakh National Medical University, Almaty 050000, Kazakhstan; ismira.gasanova.02@mail.ru; 6 School of General Medicine-2, Asfendiyarov Kazakh National Medical University, Almaty 050000, Kazakhstan; balauka.st@gmail.com; 7 Department of Chemistry, Abai Kazakh National Pedagogical University, Almaty 050000, Kazakhstan; baibolsynova89@gmail.com; 8 Department of Natural Sciences Pedagogy, School of Education and Humanities, SDU University, Kaskelen 040901, Kazakhstan; aibek.smagul@sdu.edu.kz; 9 Department of Economics and Planning, Asfendiyarov Kazakh National Medical University, Almaty 050000, Kazakhstan; zhoshieva.zh@kaznmu.kz; 10 Neonatology Department, Asfendiyarov Kazakh National Medical University, Almaty 050000, Kazakhstan; bozhbanbaeva.n@kaznmu.kz

**Keywords:** bronchopulmonary dysplasia, salivary miRNAs, biomarkers, extremely preterm infants, minimally invasive sampling, pilot study

## Abstract

Introduction: Bronchopulmonary dysplasia (BPD) remains the most significant complication of extreme prematurity, affecting long-term respiratory outcomes. Because current diagnostic criteria identify only established lesions at 36 weeks postmenstrual age, early non-invasive biomarkers are needed. This pilot study aimed to identify a candidate salivary miRNA panel associated with BPD risk and to explore its pathogenetic relevance through in silico analysis. Methods: Saliva was collected from 20 preterm infants (10 with BPD and 10 controls), and miRNA expression was profiled using the GeneChip™ miRNA 4.1 Array Plate. Discriminatory performance was explored by ROC analysis within this discovery cohort, together with power and Spearman correlation analyses. Results: Expression of hsa-let-7b-5p, hsa-let-7c-5p, and hsa-miR-4454 was significantly elevated in the BPD group (*p* < 0.05, log_2_FC ≥ 1.0), with no significant correlation with gestational age or birth weight. Bootstrap-corrected AUC values ranged from 0.905 to 0.937 and were supported by leave-one-out cross-validation. All three miRNAs showed very large effect sizes exceeding the minimum detectable effect at 80% power. Conclusions: In this pilot study, salivary miRNAs represent a hypothesis-generating candidate biomarker signal for BPD that requires external validation in larger, independent cohorts before any diagnostic or prognostic application can be considered.

## 1. Introduction

Bronchopulmonary dysplasia (BPD) remains the most significant complication associated with extreme prematurity, with lasting effects on respiratory and neuropsychiatric outcomes in extremely low-birth-weight (ELBW) and very low-birth-weight (VLBW) infants. Although “gentle” ventilation strategies and early surfactant administration have been implemented, the incidence of BPD has not consistently declined. This persistence is attributed to the increased survival of infants born during the canalicular and saccular stages of lung development. The current definition of BPD, which is based on oxygen requirements at 36 weeks postmenstrual age, identifies an already established lesion, whereas the optimal period for preventive interventions occurs much earlier [1,2,3,4].

The identification of early biomarkers for bronchopulmonary dysplasia in cord or peripheral blood is hindered by significant methodological challenges. Most circulating molecules reflect systemic responses to prematurity, sepsis, or hypoxia, thereby reducing their specificity for predicting lung tissue outcomes. Additionally, repeated blood sampling in newborns weighing less than 1000 g increases the risk of iatrogenic anemia and pain-related stress, thereby restricting opportunities for longitudinal monitoring during this critical developmental period [5].

In recent years, the concept of saliva-based “liquid biopsy” has introduced significant advancements in neonatology. Saliva collected from premature infants receiving either non-invasive or invasive respiratory support represents a unique biological reservoir. In contrast to blood, saliva may partially reflect airway-related molecular signals and represents a promising discovery matrix. However, its relationship to lung-local miRNA biology remains insufficiently established, and it should not be regarded as a direct surrogate for alveolar or interstitial biology. Moreover, while saliva collection avoids the risks associated with repeated blood sampling, obtaining an adequate volume from extremely preterm infants is technically demanding; so the method is more accurately described as minimally invasive rather than entirely non-invasive. Its relative accessibility nonetheless makes it a valuable substrate for exploring candidate biomarkers that may reflect local changes in the alveoli and interstitium [6,7].

The application of salivary biomarkers in pediatric and neonatal medicine extends well beyond BPD. Recent studies have investigated salivary molecular markers in neonatal sepsis [8], pediatric pneumonia [9], appendicitis [10], and autism spectrum disorder [11]. These studies collectively demonstrate the feasibility and diagnostic utility of saliva-based molecular profiling in pediatric populations in whom invasive sampling is not feasible, reinforcing the rationale for exploring saliva as a discovery matrix for BPD biomarkers. Beyond biomarker discovery, saliva has also served as a practical alternative to blood for therapeutic drug monitoring in preterm infants, as demonstrated nearly two decades ago in a pharmacokinetic study of theophylline for apnea of prematurity [12].

miRNAs are small noncoding RNAs that mediate post-transcriptional gene regulation and are recognized as key contributors to the pathogenesis of various diseases, including BPD. These molecules are stable in biological fluids and play a critical role in regulating angiogenesis and alveolarization, rendering them suitable candidates for non-invasive biomarker discovery in neonatal populations. A major challenge in miRNA analysis in the context of BPD is distinguishing miRNAs associated with systemic inflammation from those that directly influence lung development. Targeted analysis of specific miRNA panels enables the identification of pathways most critically involved in BPD pathogenesis, including those regulating pulmonary morphogenesis and inflammatory signaling [13,14].

Although salivary miRNAs hold considerable promise, their prognostic value remains uncertain owing to the small sample sizes reported to date. Rather than resolving this limitation, the present study, itself based on a small pilot cohort, contributes additional exploratory evidence. This is achieved through targeted salivary miRNA profiling on days 10 to 14 of life, combined with bootstrap- and cross-validation-corrected discriminative analysis and explicit, design-based reporting of statistical sensitivity, to inform the design of adequately powered future studies.

This study aimed to identify a candidate salivary miRNA panel associated with BPD risk in VLBW and ELBW preterm infants and to explore its potential pathogenetic relevance through comprehensive bioinformatics analysis as a basis for hypothesis generation and future validation.

## 2. Materials and Methods

### 2.1. Study Design and Ethical Approval

This prospective cohort study was conducted in the Neonatal Intensive Care Unit at the Center for Perinatology and Pediatric Cardiac Surgery in Almaty between January and July 2025. The study protocol received approval from the Local Ethics Committee (Protocol No. 19 (155), 30 September 2024). All procedures adhered to the ethical standards outlined in the World Medical Association’s Declaration of Helsinki. Written informed consent for participation and data processing was obtained from the legal guardians of all newborns prior to inclusion.

### 2.2. Inclusion and Exclusion Criteria

The study cohort comprised preterm infants with a birth weight below 1500 g, corresponding to the standard clinical definition of VLBW infants. Within this cohort, infants with a birth weight below 1000 g were further designated as ELBW infants. All enrolled infants were admitted to the neonatal intensive care unit (NICU) within the first 24 h of life. Infants were excluded if they were born at full term, had a birth weight greater than 1500 g, presented with verified congenital malformations, genetic abnormalities, chromosomal disorders, or experienced severe perinatal asphyxia, defined as an Apgar score below 3 at 5 min.

In addition to these enrollment criteria, several neonatal morbidities commonly observed in this population were systematically documented over the course of hospitalization using the following pre-specified, standardized diagnostic criteria, applied prospectively and uniformly to all study participants. Sepsis occurring within the first 72 h of life was classified as early-onset, and sepsis occurring thereafter was classified as late-onset. Sepsis was diagnosed on the basis of clinical signs of systemic infection together with a positive blood and/or cerebrospinal fluid culture, supported where necessary by elevated C-reactive protein and/or procalcitonin, an abnormal leukocyte count, or an elevated immature-to-total neutrophil ratio [15,16]. Necrotizing enterocolitis (NEC) was diagnosed and staged according to the modified Bell’s staging criteria, with a diagnosis of NEC requiring stage IIA or higher, defined by pneumatosis intestinalis on abdominal radiography together with compatible clinical signs [17,18,19]. Disseminated intravascular coagulation (DIC) was diagnosed using a scoring system based on platelet count, prothrombin time, and fibrinogen/D-dimer levels, consistent with the International Society on Thrombosis and Haemostasis criteria for overt DIC [20]. Intraventricular hemorrhage (IVH) was graded by cranial ultrasonography according to the classification of Papile et al., ranging from Grade I, defined as subependymal hemorrhage, to Grade IV, defined as intraparenchymal extension [21]. Periventricular leukomalacia (PVL) was diagnosed by serial cranial ultrasonography according to the criteria of de Vries et al., on the basis of the presence and evolution of periventricular echodensities and echolucencies [22]. Anemia of prematurity was defined according to postnatal age-adjusted hemoglobin and hematocrit reference thresholds [23]. Retinopathy of prematurity (ROP) was screened for and staged according to the International Classification of Retinopathy of Prematurity, Third Edition [24].

### 2.3. Outcomes of the Study

Primary outcome: identification of differentially expressed salivary miRNAs (|log_2_FC| ≥ 1.0, FDR < 0.05) between preterm infants who subsequently developed BPD (BPD+) and those who did not (BPD−).

Secondary outcomes: (1) evaluation of the individual discriminatory performance of candidate miRNAs using ROC curve analysis; (2) functional annotation of miRNA target genes through Gene Ontology Biological Process (GO-BP) and Kyoto Encyclopedia of Genes and Genomes (KEGG) pathway enrichment analyses; and (3) descriptive characterization of miRNA expression patterns across BPD and control samples.

### 2.4. Patient Recruitment and Stratification

The clinical status of newborns was assessed daily from birth until discharge or transfer to the next stage of intensive care. Infants were classified as BPD or non-BPD based on their respiratory status at 36 weeks’ postmenstrual age, in accordance with the framework proposed by Higgins et al. [25]. Based on this endpoint, patients were divided into two groups. The main group, designated BPD+ (*n* = 10), comprised infants who required supplemental oxygen or respiratory support at 36 weeks’ postmenstrual age. The comparison group, designated BPD− (*n* = 10), comprised infants who did not require supplemental oxygen or respiratory support at 36 weeks’ postmenstrual age.

**Figure 1:**
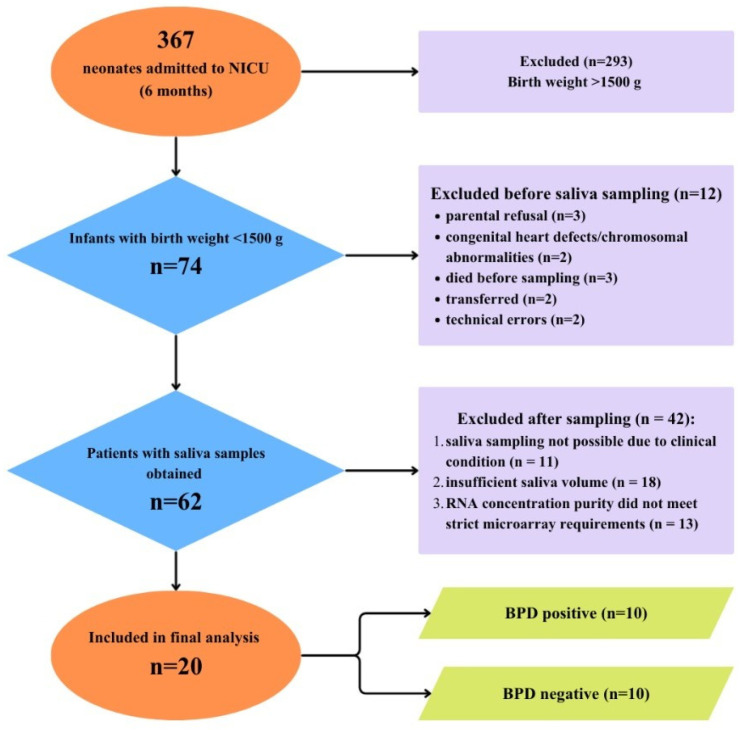
Stages in the formation of the study groups based on patient selection criteria.

The patient selection algorithm, including the inclusion, exclusion, and final stratification stages, is presented in the flowchart in Figure 1.

### 2.5. Collection of Saliva Samples and Their Transportation

Biological samples were obtained from premature infants to facilitate total RNA extraction and transcriptome analysis. All procedures used aseptic, minimally invasive techniques.

Saliva samples were collected on postnatal days 10–14, one hour prior to feeding to minimize the impact of dietary nutrients on the miRNA profile. Two collection methods were initially evaluated: absorption with a sterile cotton swab and aspiration with a sterile syringe. The pilot phase revealed that cotton swabs yielded insufficient biomaterial volumes due to physiologically low salivary secretion in premature infants. Consequently, all subsequent samples were collected exclusively by aspiration. No pharmacological or mechanical sialogogue methods were used to stimulate salivary secretion. Saliva was therefore collected passively from resting secretion to preserve the native salivary microRNA profile and minimize potential alterations in transcriptomic composition.

This conservative, unstimulated approach, together with the clinical fragility of the study population, contributed to a substantial sample failure rate. Saliva sampling could not be attempted in 11 infants due to their underlying clinical condition. Both invasive and non-invasive respiratory support dried the oral mucosa and necessitated frequent oral suctioning, further limiting salivary output. Enteral feeding was frequently withheld in infants with suspected sepsis, and fluid intake was restricted in those with a hemodynamically significant patent ductus arteriosus, both of which reduced salivary output. Impaired tissue perfusion secondary to shock or DIC similarly limited exocrine secretion. Sedative and analgesic use further diminished autonomic salivary stimulation. The limited oral cavity volume characteristic of ELBW infants additionally constrained sample collection. In a further 18 of the 62 infants initially screened, sampling was attempted but yielded an insufficient volume, consistent with the physiologically low, unstimulated salivary output expected in this population (Figure 1).

Saliva was collected from the oral cavity of newborns using a sterile syringe and subsequently transferred into sterile cryogenic tubes. Samples were collected by a dedicated team of trained NICU nurses following a written standard operating procedure to reduce inter-operator variability. Due to the anatomical and physiological characteristics of premature infants, including immature salivary glands and reduced salivation, the average sample volume obtained was 0.3 mL.

Immediately following collection, RNAlater stabilizing reagent (Thermo Fisher Scientific, Waltham, MA, USA) was added to each sample. Owing to the limited volume of biomaterial, a 1:1 saliva-to-reagent ratio was maintained (0.3 mL saliva and 0.3 mL RNAlater). The tubes were sealed and gently shaken for 10 to 15 s to ensure uniform RNA stabilization. Prior to transport, samples were stored at 4 °C for short-term preservation. Subsequently, samples were transported in a long-term storage container for two hours before being placed in a −80 °C freezer until laboratory analysis. Sterile conditions were rigorously maintained throughout all procedures to prevent RNA degradation and sample contamination.

### 2.6. Extraction of miRNA from Saliva

Prior to extraction, the stabilizing solution was removed, and the samples were subsequently pre-cleared. A volume of 100 µL of processed saliva served as the input for RNA extraction. Total RNA, including the miRNA fraction, was isolated using the MagMAX™ mirVana™ Total RNA Isolation Kit (Thermo Fisher Scientific, Waltham, MA, USA).

During the pre-treatment step, 5 µL of Proteinase K was added to each well of a KingFisher 96 Deep-Well Plate (Thermo Fisher Scientific, Waltham, MA, USA), followed by 100 µL of saliva sample and 45 µL of PK Digestion Buffer. The mixture was shaken for 5 min at 950 rpm and then incubated for 30 min at 65 °C. This procedure facilitated efficient proteolysis and prepared the samples for subsequent lysis.

Samples were lysed, and RNA was adsorbed onto magnetic particles by mixing 99 µL of Lysis Buffer with 1 µL of 2-mercaptoethanol per sample. Each sample received 100 µL of this mixture and 20 µL of RNA Binding Beads, followed by shaking for 7 min at 700 rpm. Subsequently, 270 µL of isopropanol was added, mixed by pipetting, and incubated with shaking to facilitate RNA binding to the magnetic particles.

Magnetic particle washing was conducted using a Magnetic Stand-96 (Thermo Fisher Scientific, Waltham, MA, USA). Following sedimentation and removal of the supernatant, 150 µL of Wash Solution 1 and subsequently 150 µL of Wash Solution 2 were sequentially added to the samples, with each step involving shaking and magnetic separation. After washing, the particles were dried in an open plate for 5 min at 1150 rpm. Care was taken to avoid overdrying, as this could reduce the efficiency of subsequent elution.

Contaminating DNA was removed by treating the samples with 50 µL of TURBO DNase™ Solution, followed by incubation for 15 min at 1050 rpm. RNA was rebound to magnetic beads through the sequential addition of 50 µL of Rebinding Buffer and 100 µL of isopropanol, without pre-mixing. The plate was shaken for 5 min at 950 rpm. Magnetic separation was performed, followed by two washes with Wash Solution 2 (150 µL per sample).

Final RNA elution was achieved by adding 50 µL of pre-warmed Elution Buffer to the dried magnetic particles. Samples were vortexed for 2 min at 1050 rpm, incubated for 5 min at 65 °C, and vortexed again for 2 min at 1050 rpm. After magnetic separation, the supernatant containing purified RNA was transferred to an Elution Plate. The RNA preparations were either used immediately, stored on ice for up to 8 h, or kept at −20 °C for extended storage.

### 2.7. Biotin Labeling of miRNAs and Preparation for Hybridization

RNA samples were labeled with the FlashTag™ Biotin HSR RNA Labeling Kit (Thermo Fisher Scientific, Waltham, MA, USA) following the manufacturer’s protocol. The procedure involves a sequential poly(A) tailing reaction followed by ligation of a biotinylated signal molecule to the target RNA, enabling sensitive and reproducible labeling of low-molecular-weight RNAs.

The RNA sample volume was adjusted to 8 µL with nuclease-free water. Subsequently, 2 µL of RNA Spike Control Oligos was added, and the mixture was maintained on ice. ATP Mix was pre-diluted in 1 mM Tris. For total RNA samples, a 1:500 dilution was applied. For enriched and quantified samples, the ATP Mix dilution factor was determined using the formula: 5000 divided by the amount of LMW RNA added (ng). For enriched samples without quantification, the dilution factor was calculated as 1000 divided by the amount of total RNA added (µg).

A master mix for the poly(A) tailing reaction was prepared in a nuclease-free tube. Each reaction included 1.5 µL of 10× Reaction Buffer, 1.5 µL of 25 mM MnCl_2_, 1.0 µL of pre-diluted ATP Mix, and 1.0 µL of PAP Enzyme. Five microliters of the master mix were added to 10 µL of the RNA/Spike Control Oligos mixture, resulting in a total reaction volume of 15 µL. Following gentle mixing and brief centrifugation, the samples were incubated at 37 °C for 15 min.

The ligation reaction was performed immediately after the poly(A) tailing step. Samples were briefly centrifuged and placed on ice, after which 4 µL of 5× FlashTag Biotin HSR Ligation Mix and 2 µL of T4 DNA Ligase were added to each sample individually rather than as a master mix to prevent autoligation. After gentle mixing and brief centrifugation, the reaction was incubated at 25 °C for 30 min. The reaction was terminated by the addition of 2.5 µL of HSR Stop Solution, yielding a final volume of 23.5 µL per sample. Before hybridization, samples were stored on ice for up to 6 h or at −20 °C for up to 2 weeks.

Quality control was implemented at two stages of sample preparation. At the labeling stage, the inclusion of RNA Spike Control Oligos in each sample allowed independent monitoring of biotin labeling and ligation efficiency, irrespective of biological material quality. At the hybridization stage, the built-in hybridization and spike controls of the GeneChip™ miRNA 4.1 Array Plate platform (Thermo Fisher Scientific, Waltham, MA, USA) were used to verify the accuracy of the hybridization, washing, and scanning procedures. Control metrics were assessed in Expression Console™ software, and all samples passed predefined quality thresholds before downstream normalization and differential expression analysis.

### 2.8. Hybridization and Expression Analysis of miRNAs on the Genechip^TM^ miRNA 4.1 Array Plate Platform

After biotin labeling of miRNAs with the FlashTag™ Biotin HSR RNA Labeling Kit, samples were analyzed for miRNA expression using GeneChip™ miRNA 4.1 Array Plate microarrays, which accommodate up to 96 samples simultaneously. This platform offers comprehensive coverage of mature miRNA sequences annotated in miRBase and is optimized for high-throughput analysis of small non-coding RNA expression profiles.

Before hybridization, hybridization ovens, microarrays, and sample log files were prepared using GeneChip™ Command Console software (version 6.1.0.190) according to the manufacturer’s recommendations. Biotinylated RNA samples were combined to create a hybridization mixture containing hybridization buffer, formamide, DMSO, and standard controls. This mixture was denatured and incubated at controlled temperatures to promote efficient binding of labeled miRNAs to the microarray oligonucleotide probes.

Hybridization was conducted automatically using a GeneTitan™ instrument (Thermo Fisher Scientific, Waltham, MA, USA), followed by washing and staining with the GeneTitan™ Hybridization, Wash, and Stain Kit for miRNA Array Plates. All microarray processing steps adhered to the recommended protocols and temperature conditions to ensure reproducibility and minimize technical variation.

Microarray scanning was conducted automatically, resulting in the generation of raw data files (CEL files). Experimental quality control utilized built-in hybridization and spike controls to assess the accuracy of labeling, hybridization, and scanning procedures. Primary processing, normalization, and quality control of miRNA expression data were conducted using Expression Console™ software (version 1.4.1) with the RMA plus DABG algorithm. The processed data were then exported for subsequent statistical analysis.

### 2.9. Bioinformatic Analysis

Raw Affymetrix miRNA CEL files containing complete microarray data were imported using the oligo package (v1.74.0) and normalized with the Robust Multi-array Average (RMA) method to generate log_2_-transformed expression values. Probe sets were annotated with the miRNA-4_1-st-v1 reference file (v20160922). Annotation entries were filtered to retain only Homo sapiens entries with the sequence type “miRNA.” Probes with a mean log_2_ intensity of 5 or less across all samples were excluded to reduce technical noise. Principal component analysis (PCA) was used for sample-level quality control. Differential miRNA expression between BPD and control groups was assessed using limma (v3.66.0) with a two-group design matrix encoding condition membership, empirical Bayes moderation via eBayes, and Benjamini–Hochberg FDR correction. miRNAs with |log_2_FC| ≥ 1 and FDR < 0.05 were considered differentially expressed and visualized as volcano plots. For each significantly differentially expressed miRNA, individual receiver operating characteristic (ROC) curves were generated using pROC (v1.19.0.1), and the area under the curve (AUC) with its 95% confidence interval was estimated. miRNAs with an AUC of 0.75 or greater were retained as final biomarker candidates. The optimal classification cutoff for each miRNA was selected by the Youden index on the RMA-normalized log_2_ expression scale, with values greater than or equal to the cutoff classified as BPD. To address optimistic bias inherent in discovery-set AUC estimation, bootstrap-corrected AUC (1000 iterations) and leave-one-out cross-validation (LOOCV) AUC were additionally computed for each final candidate using the Efron–Tibshirani optimism-correction method [26]. Importantly, this LOOCV was applied only to the classification step for the three pre-selected candidates. Validated miRNA-mRNA interactions for the candidate miRNAs were obtained using the multiMiR package (v1.32.0) by querying validated interaction tables, and unique target gene symbols were compiled. Functional enrichment analysis of validated targets was conducted using clusterProfiler (v4.18.4), separately for GO-BP terms and KEGG pathways. To characterize the regulatory network, validated miRNA-mRNA interaction pairs were filtered to the 100 most highly connected target genes, ranked by the number of targeting miRNAs (hub degree). The resulting directed sub-network was exported for downstream visualization in Gephi (v0.10.1). Spearman rank correlations between each candidate miRNA and gestational age and birth weight were computed within the BPD-positive group. Raw and processed microarray data have been deposited in the NCBI Gene Expression Omnibus (GEO) under accession number GSE336455.

### 2.10. Statistical Analysis

Prior to analysis, all continuous variables were assessed for normality using the Shapiro–Wilk and Kolmogorov–Smirnov tests. All continuous variables demonstrated non-normal distributions (*p* < 0.05). Therefore, baseline characteristics are summarized as medians with interquartile ranges (IQRs) for continuous variables and as counts with percentages for categorical variables. Group comparisons employed the Wilcoxon rank-sum test for continuous variables and Fisher’s exact test for categorical variables. No missing values were detected. All analyses were performed in R (v4.5.1), and a two-sided *p*-value < 0.05 was deemed statistically significant unless otherwise specified. The sensitivity of the design was characterized by the minimum detectable effect (MDE), a prospective, design-based quantity, rather than by post hoc power, which is a deterministic function of the *p*-value and therefore uninformative [27]. For a two-sided Wilcoxon rank-sum test with *n* = 10 per group, α = 0.05, and 80% power, the MDE was Cohen’s d = 1.519. The design was thus sensitive only to very large effects and underpowered for smaller ones. Observed effect sizes are reported descriptively and are not used to infer adequate power.

## 3. Results

### 3.1. Clinical Characteristics of the Study Group

Clinical and demographic characteristics differed significantly between the BPD+ and BPD− groups. BPD+ infants were born earlier and at a lower birth weight, with a correspondingly more complicated neonatal course, findings consistent with the established epidemiology of BPD. The BPD+ group additionally exhibited substantially higher rates of several neonatal morbidities, including sepsis, NEC, and DIC. The potential confounding effects of these concurrent morbidities on miRNA expression could not be statistically evaluated owing to the limited sample size. At 36 weeks postmenstrual age, disease severity within the BPD+ group was classified as mild in 3 patients, moderate in 4 patients, and severe in 3 patients. Detailed statistical parameters describing the distribution of these and other clinical characteristics across the study cohort are presented in Table 1.

### 3.2. Global Expression Patterns and Quality Control

**Figure 2:**
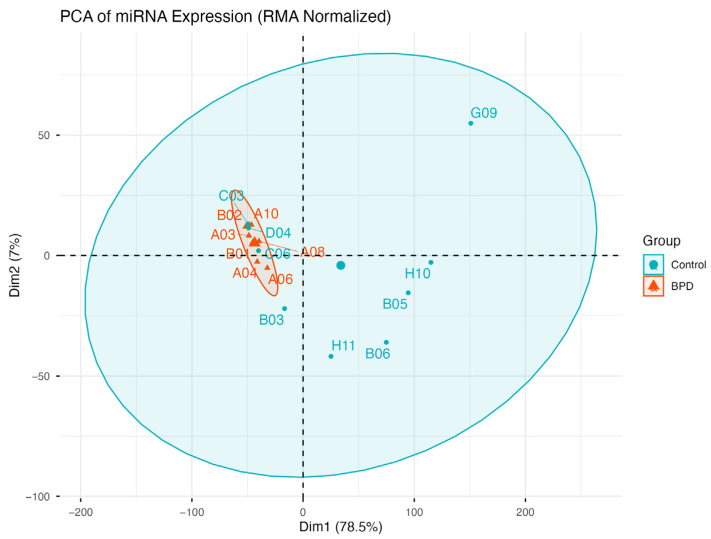
Principal Component Analysis for quality control of miRNA expression data. Each point corresponds to an individual sample: orange triangles represent infants with bronchopulmonary dysplasia (BPD+, *n* = 10), and blue circles represent control infants without BPD (BPD−, *n* = 10). Dim1 (PC1) and Dim2 (PC2) explain 78.5% and 7% of the total variance, respectively. BPD+ samples form a compact cluster with low within-group variability, restricted to the negative region of Dim1. Control samples exhibit substantially greater within-group dispersion across both dimensions, with three control samples (C06, C03, D04) located near the BPD+ cluster. The 95% confidence ellipse for the BPD+ group indicates high transcriptomic homogeneity. No equivalent ellipse is provided for the control group because its distribution is heterogeneous. Complete separation between the groups was not observed. Abbreviations: PCA, principal component analysis; RMA, Robust Multi-array Average; BPD+, bronchopulmonary dysplasia group; BPD−, control group; Dim, dimension.

Principal component analysis was performed to evaluate the effectiveness of data normalization via the RMA method and to characterize global transcriptomic variation across samples (Figure 2). The first principal component (PC1) accounted for 78.5% of the total variance, while the second principal component (PC2) explained an additional 7%.

BPD+ samples formed a compact cluster, indicating low within-group variability. In contrast, control samples exhibited substantially greater within-group dispersion, with three control samples located near the BPD+ cluster. Complete separation between the groups was not observed.

### 3.3. Differential Expression Profiling of Human miRNAs in BPD Patients

Differential expression analysis identified 197 miRNAs exhibiting differential expression across the study groups. To reduce the likelihood of false-positive findings, Benjamini–Hochberg correction for multiple testing was applied. Three miRNAs remained significant at an adjusted *p*-value threshold of 0.05. The detailed expression statistics for these three key miRNAs, including log fold changes and adjusted *p*-values, are summarized in Table 2.

**Figure 3:**
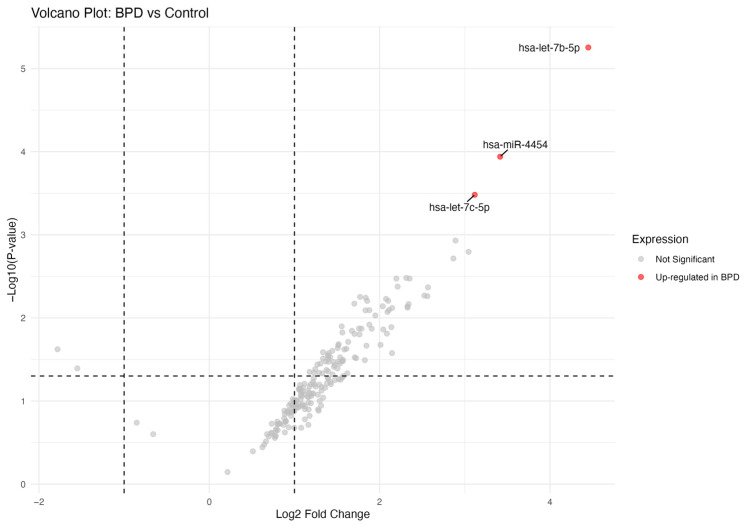
Volcano plot of differentially expressed miRNAs between the BPD and non-BPD groups. The x-axis displays the log_2_ fold change (log_2_FC) in expression between the BPD+ and BPD− groups, while the y-axis shows the −log_10_-transformed Benjamini–Hochberg-adjusted *p*-value. Each point corresponds to a single miRNA probe. Red circles denote miRNAs that meet both significance thresholds: adjusted *p*-value < 0.05 (horizontal dashed line) and |log_2_FC| ≥ 1.0 (vertical dashed lines). Gray circles represent miRNAs that do not meet these criteria. Abbreviations: log_2_FC, log_2_ fold change; adj. *p*, Benjamini–Hochberg-adjusted *p*-value.

Among these, hsa-let-7b-5p showed the lowest adjusted *p*-value (adj. *p* = 0.001) and the greatest fold change (log_2_FC = 4.45), making it the most statistically compelling candidate. hsa-miR-4454 and hsa-let-7c-5p were also significantly upregulated, with adjusted *p*-values of 0.011 and 0.021, respectively, and fold changes of 3.41 and 3.12. All three molecules were consistently upregulated in the BPD+ group. The overall distribution and statistical significance of the full analysis are visually demonstrated in the volcano plot (Figure 3). Data for the full set of 197 differentially expressed molecules are provided in Supplementary Table S1.

### 3.4. Hierarchical Clustering Analysis

**Figure 4:**
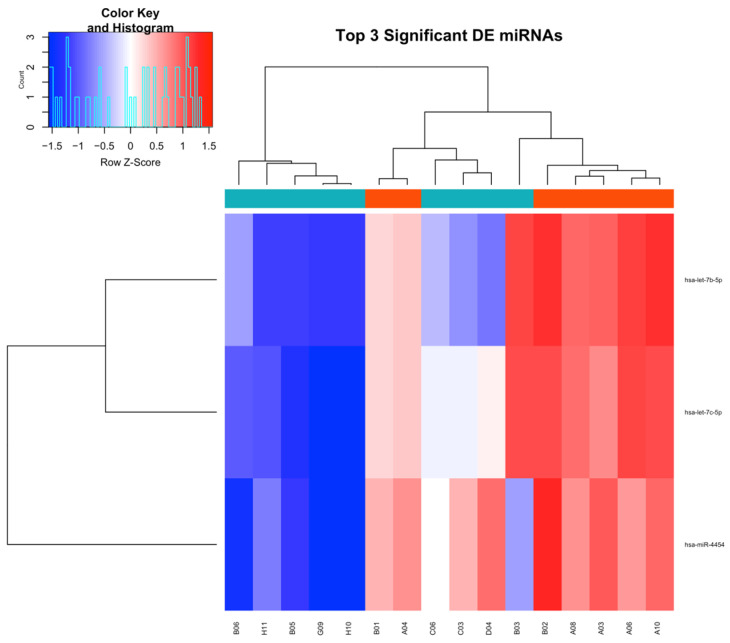
Hierarchical clustering heatmap of the top 3 differentially expressed miRNAs. Rows correspond to individual miRNAs (hsa-let-7b-5p, hsa-let-7c-5p, hsa-miR-4454), while columns represent individual samples. The color scale indicates row-normalized Z-scores, with red denoting higher expression and blue denoting lower expression. The color bar above the heatmap indicates group membership: teal for the control (BPD−) and orange-red for BPD+ samples. Dendrograms on both axes were generated using complete-linkage hierarchical clustering. The inset histogram shows the distribution of Z-scores across all displayed probes. Abbreviations: DE, differentially expressed; Z-score, row-normalized expression value.

Hierarchical clustering was performed to evaluate the expression consistency of the three identified miRNAs across individual samples. The heatmap (Figure 4) showed markedly elevated expression of hsa-let-7b-5p, hsa-let-7c-5p, and hsa-miR-4454 in most BPD+ samples, while controls generally maintained low expression levels across all three molecules.

Complete group separation was not achieved, as the BPD+ and BPD− samples were partially interleaved in the dendrogram.

### 3.5. Descriptive Association Between Candidate miRNA Expression and Gestational Age and Birth Weight

Gestational age and birth weight were strongly associated with BPD status in this cohort and are established correlates of BPD risk. As a descriptive summary, the rank associations between each candidate miRNA and gestational age or birth weight was quantified using Spearman coefficients (Table 3). None reached nominal significance (*p* > 0.05 for all comparisons). A bivariate correlation between a marker and a covariate is not a test of confounding as it does not evaluate whether the covariate distorts the miRNA–BPD association. Because gestational age and birth weight differ substantially between groups and are collinear with group membership, and because the sample is small, the independent contribution of the candidate miRNAs cannot be separated from that of prematurity.

### 3.6. Individual Discriminatory Performance of Candidate miRNAs

Receiver operating characteristic (ROC) analysis was conducted to evaluate the discriminatory performance of each miRNA individually within this discovery cohort (Table 4). The bootstrap area under the curve (AUC) values were 0.937 (95% CI 0.780–1.000) for both hsa-let-7b-5p and hsa-miR-4454, and 0.905 (95% CI 0.680–1.000) for hsa-let-7c-5p. At the optimal cutoff values, all three markers achieved 100% sensitivity and 90.0% specificity, with a positive predictive value of 90.9% and a negative predictive value of 100%. Exact 2 × 2 contingency tables for each miRNA, including the raw true-positive, false-positive, false-negative, and true-negative counts, are provided in Supplementary Table S2. The partial AUC, constrained to a minimum specificity of 80%, was 0.824 for hsa-let-7b-5p and hsa-miR-4454 and 0.735 for hsa-let-7c-5p. Leave-one-out cross-validation (LOOCV) AUC values were 0.944 for hsa-let-7b-5p and 0.833 for both hsa-miR-4454 and hsa-let-7c-5p.

### 3.7. Statistical Power Analysis

A design-based sensitivity analysis was conducted to characterize which effect magnitudes that this sample size could detect, using the minimum detectable effect (MDE). For a two-sided Wilcoxon rank-sum test with α = 0.05 and 80% power, the smallest detectable standardized effect was Cohen’s d = 1.519—a very large effect by conventional benchmarks. The study was therefore sensitive only to very large between-group differences and was underpowered to detect small or moderate effects, any of which would likely have gone undetected. Observed effect sizes are shown in Table 5 for descriptive purposes only. Because they were estimated from the same small sample that was selected for statistical significance, they are expected to be upwardly biased and are not used to infer that the study was adequately powered.

### 3.8. Functional Annotation and Pathway Enrichment Analysis of miRNA Target Genes

To clarify the molecular mechanisms underlying the discriminatory performance of hsa-let-7c-5p, hsa-miR-4454, and hsa-let-7b-5p, an in silico analysis was performed to identify their genetic targets, resulting in 27,049 unique validated genes. The complete list of genes is provided in Supplementary Table S3.

**Figure 5:**
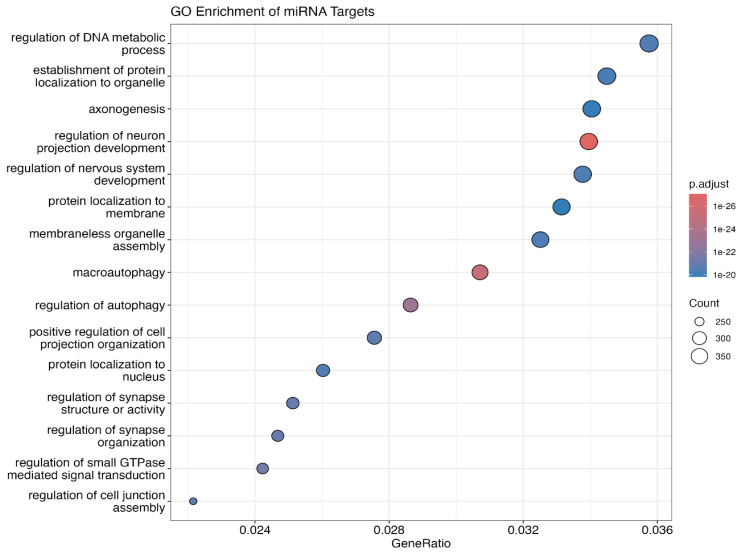
Biological processes associated with the identified salivary miRNA panel in extremely preterm infants. The diagram displays the results of enrichment analysis by the “Biological Processes” category. The dot size correlates with the number of target genes involved in the corresponding process, and the color scale indicates the level of statistical significance.

Gene ontology enrichment analysis identified 15 significantly enriched biological process terms among the predicted targets of the three miRNAs (Figure 5). The highest GeneRatio was observed for the regulation of the DNA metabolic process, which also had the largest number of associated genes. Establishment of protein localisation to organelles was the second-most enriched term by GeneRatio.

Three of the five most enriched terms were related to neurological function, namely axonogenesis, regulation of neuron projection development, and regulation of nervous system development. Among all enriched terms, regulation of neuron projection development showed the lowest adjusted *p*-value and was therefore the most statistically significant finding in the analysis.

Autophagy-related terms were also represented, with macroautophagy and regulation of autophagy both reaching significance. Further enriched processes included protein localisation to membrane, membraneless organelle assembly, protein localisation to the nucleus, and regulation of cell junction assembly.

**Figure 6:**
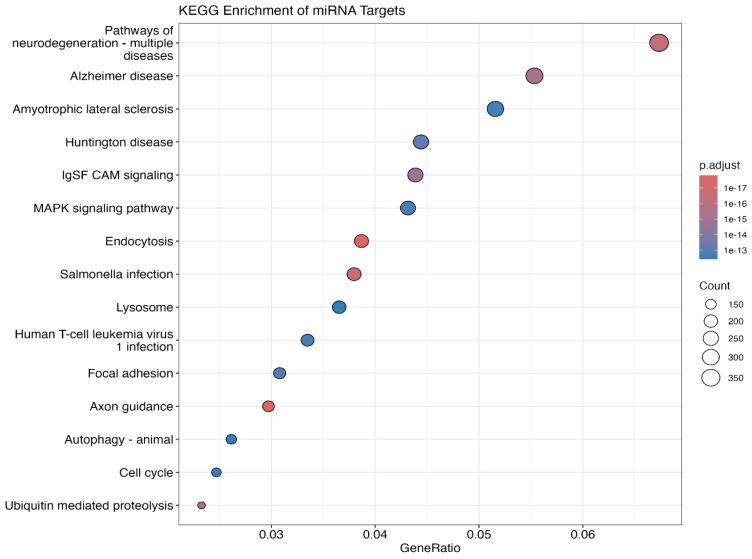
Functional annotation of the target genes of the prognostic miRNA panel using KEGG pathway enrichment analysis. The dot plot presents the most statistically significant signaling pathways associated with the target genes of the differentially expressed miRNAs. Dot size represents the proportion of genes from the analyzed set involved in each signaling cascade, while the color gradient denotes the level of statistical significance.

KEGG pathway enrichment analysis was performed to further characterize the biological pathways represented among the predicted targets of the three miRNAs (Figure 6). Among the most significantly enriched terms were cell cycle regulation, ubiquitin-mediated proteolysis, and autophagy. Lysosomal and endocytic pathways were also enriched, consistent with the broad overrepresentation of target genes in intracellular trafficking and protein quality control processes. MAPK signaling, focal adhesion, axon guidance, and cell adhesion molecule signaling were additionally enriched. The recurrence of axon guidance and neuronal signaling terms in the KEGG output mirrors the pattern observed in the GO enrichment analysis and most likely reflects the same underlying target gene set rather than an independent biological signal. Several neurodegenerative disease entries appeared in the enrichment output, including Huntington’s disease, Alzheimer’s disease, and Parkinson’s disease.

### 3.9. Network Analysis of Interactions Between miRNAs and Target Genes

**Figure 7:**
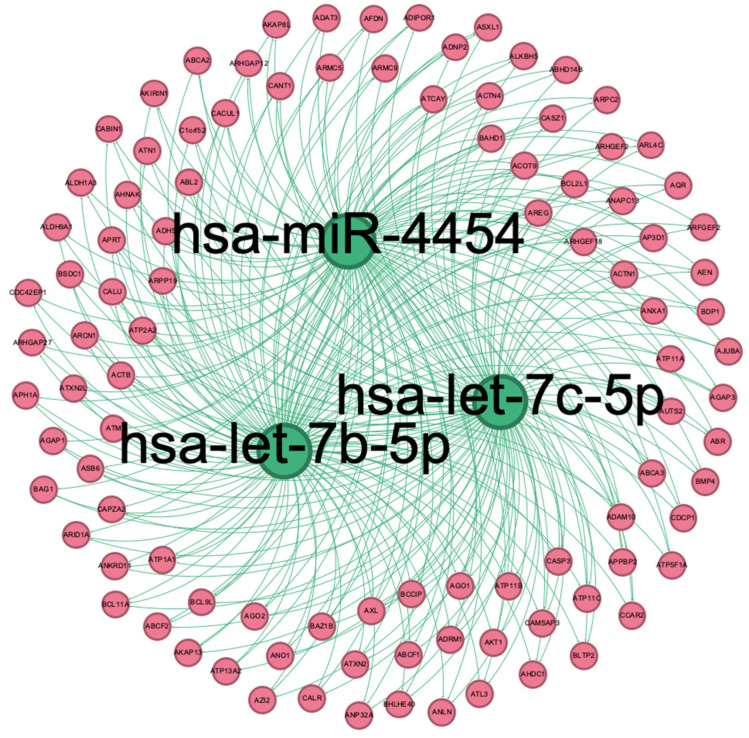
Integrated miRNA–target gene interaction network for the predictive salivary biomarker panel. The presented interactome demonstrates the regulatory architecture between differentially expressed miRNAs and their validated mRNA targets. Large diamond-shaped nodes represent miRNAs, and small round nodes represent target genes.

An miRNA–target interaction network was constructed to map the regulatory relationships between the three miRNAs and their predicted target genes (Figure 7). All three miRNAs appear as central hub nodes, each connected to a large number of peripheral target genes. The network structure is consistent with the broad regulatory scope suggested by the target gene counts reported above.

Visually, hsa-let-7b-5p and hsa-let-7c-5p appear to share a substantial proportion of their targets, which is expected given their membership in the same miRNA family. hsa-miR-4454 shows a partially overlapping but distinct target set. Among the visible target genes, several are of particular biological relevance in the context of BPD. *BCL2L1* and *CASP3* are both present, implicating apoptotic regulation among the predicted downstream effects. *AKT1*, a central node in PI3K-Akt signaling, is also visible, consistent with the KEGG enrichment findings. *AGO1* and *AGO2*, components of the RNA-induced silencing complex, appear as targets, suggesting potential feedback regulation of the miRNA machinery itself.

Several targets warrant specific mention in the context of lung development and injury. *AREG* encodes amphiregulin, a ligand involved in epithelial repair and airway remodeling. *BMP4* is a key regulator of alveolar development. *ATM* is a central mediator of the DNA damage response.

## 4. Discussion

This pilot study identifies a candidate salivary miRNA panel, comprising hsa-let-7b-5p, hsa-let-7c-5p, and hsa-miR-4454, the expression of which was significantly elevated in extremely preterm infants who subsequently developed bronchopulmonary dysplasia relative to those who did not. These findings are exploratory and should be considered in light of the study’s small, single-center cohort and notable intergroup differences in gestational age and birth weight. The mechanistic associations discussed below are based on previously published experimental studies and are provided for biological context only. They do not represent mechanistic evidence generated by the present investigation.

The role of miRNAs in lung development and the pathogenesis of BPD has been established in multiple studies [1,10,11]. However, most investigations have focused on miRNAs in serum or tracheal aspirates, leaving a notable scarcity of data on miRNAs in saliva from patients with BPD. Saliva was chosen as the study matrix due to its bioavailability and the ability of salivary exosomes to transport miRNAs that may reflect respiratory system status. This selection is supported by the close embryological relationship between the oral cavity and the airways, as well as their ongoing aspiration contact. This rationale is consistent with the hypothesis that circulating miRNAs act as mediators of intercellular communication across distant sites, transmitting signals of airway epithelial injury to other biological fluids [28]. Maron demonstrated that the neonatal salivary transcriptome is detectable in sufficient quantities even in extremely immature neonates and may reflect local biological processes [6]. The non-invasive nature of salivary sampling is particularly important in the ELBW and VLBW populations, where repeated blood draws carry significant risks of iatrogenic anemia and procedural pain [5].

The oral microbiome is influenced by factors unrelated to BPD pathogenesis, such as feeding practices, gestational age, and mode of delivery [29,30]. Employing a rigorous preanalytical protocol to minimize contamination with non-target molecules facilitated the identification of specific changes in miRNA expression that might otherwise be masked by background noise. The most direct empirical support for saliva partially reflecting airway-related miRNA signals, rather than serving as a validated surrogate matrix, comes from Siddaiah et al. They reported a Spearman correlation (r = 0.97) between salivary and tracheal aspirate miRNA expression profiles at three days of age in seven extremely low gestational age newborns [7]. Given this very small sample, the finding should be regarded as preliminary evidence of biological plausibility rather than proof that saliva can substitute for airway sampling. Additionally, Lal et al. demonstrated that exosomal miRNAs in tracheal aspirates from extremely preterm infants, including let-7 family members, predict severe BPD and exert protective biological effects in animal models [9]. Siddaiah et al. also identified let-7 family members in the salivary transcriptome of extremely low gestational age newborns [7], while Ambalavanan et al. reported, in a conference abstract, preliminary findings proposing hsa-let-7b-5p as a candidate BPD biomarker and therapeutic target across blood, tracheal aspirate, and lung tissue samples [10]. As this work has not yet undergone full peer review, we treat it as supportive rather than confirmatory evidence. Therefore, the increased expression of hsa-let-7b-5p in the current salivary dataset is directionally consistent with findings from other biological matrices. However, BPD status in this cohort is closely tied to gestational age, birth weight, and concurrent morbidity. These factors cannot be statistically separated from BPD in the present design. This concordance therefore cannot be attributed specifically to BPD biology rather than to prematurity and its complications more broadly. In contrast, hsa-miR-4454 is a novel exploratory salivary candidate in this dataset. Its mechanistic relationship to BPD and NF-κB signaling remains hypothetical and requires replication in independent human cohorts and functional validation in human neonatal airway models.

A key issue concerns whether the identified miRNA signal reflects BPD pathobiology or merely the degree of prematurity, given that infants with BPD were born at substantially lower gestational ages and with lower birth weights (Table 1). We examined Spearman correlations between each candidate miRNA and gestational age or birth weight (Table 3). Although none reached significance (e.g., hsa-let-7b-5p and hsa-let-7c-5p versus gestational age, ρ = −0.704, *p* = 0.077; hsa-miR-4454, ρ = −0.111, *p* = 0.812), we recognize that such bivariate correlations do not constitute a test of confounding and cannot establish that the signal is independent of prematurity. Assessing confounding would require evaluating whether these covariates distort the miRNA–BPD association, which a marker–covariate correlation does not address. Moreover, the coefficients were computed within a small, group-restricted sample, which attenuates them toward the null. Gestational age and birth weight are tightly linked to BPD status and are effectively collinear with group membership in this cohort. Comorbidities such as necrotizing enterocolitis, disseminated intravascular coagulation, and posthaemorrhagic anemia also differ markedly between groups. As a result, the independent contribution of the candidate miRNAs cannot be disentangled from prematurity and its associated morbidities with the present data. We therefore do not claim that the miRNA signal is independent of prematurity. Adequately evaluating confounding will require a substantially larger, gestational age- and weight-matched (or propensity-adjusted) design. Given this pattern of concurrent morbidities, the present study design cannot determine whether the identified miRNAs are specific predictors of BPD pathobiology or, alternatively, markers of a broader neonatal inflammatory state driven by prematurity, NEC, or related complications. Future studies should incorporate disease-specific comparator groups, such as extremely preterm infants with sepsis or NEC without BPD, as well as propensity score-adjusted multivariable models in adequately powered cohorts to disentangle these effects.

The discriminatory performance of the identified miRNA panel was assessed using ROC curve analysis. hsa-let-7b-5p demonstrated a bootstrap-corrected AUC of 0.937 (95% bootstrap confidence interval [CI]: 0.780–1.000), hsa-miR-4454 demonstrated a bootstrap-corrected AUC of 0.937 (95% bootstrap CI: 0.770–1.000), and hsa-let-7c-5p demonstrated a bootstrap-corrected AUC of 0.905 (95% bootstrap CI: 0.680–1.000). At the optimal cutoffs for each candidate, sensitivity was 100% and specificity was 90.0%. Confidence intervals were estimated using 1000 bootstrap resampling iterations to account for internal variability in the AUC estimates. Leave-one-out cross-validation yielded AUC values of 0.944, 0.833, and 0.833, respectively, supporting the internal consistency of these results. Nevertheless, these performance metrics should be interpreted with caution because they are based on the discovery cohort and require external validation. The present analysis is also limited to diagnostic discrimination, evaluating only whether these candidate miRNAs distinguish BPD from control status above chance, as assessed by the area under the curve, rather than providing a calibrated risk prediction model. Calibration analysis would become relevant only if future studies advance these candidates toward individual risk prediction applications. To characterize the sensitivity of the design given the small sample, we report the minimum detectable effect rather than post hoc power. At the present group sizes (α = 0.05, 80% power), only effects as large as Cohen’s d = 1.519 or greater were detectable, so the study was well positioned to detect very large differences but underpowered for small-to-moderate ones. We deliberately avoid the circular inference of comparing the observed effect sizes—estimated from the same data and selected for significance, and therefore likely inflated—against this threshold to claim adequate power [27]. The high observed effect sizes should thus be regarded as exploratory and in need of independent confirmation, not as evidence that the study was adequately powered.

Among the three candidate miRNAs, hsa-let-7b-5p exhibited the highest fold change (log_2_FC = 4.45) and the most significant adjusted *p*-value (adj. *p* = 0.001). The biological plausibility of hsa-let-7b-5p as a BPD-associated marker is supported by evidence from target gene biology, developmental lung miRNA profiling, and multi-matrix clinical data. Ambalavanan et al. reported a 46-fold elevation of hsa-let-7b-5p in the blood of preterm infants who later developed severe BPD compared to those who did not, as well as a 14-fold increase in tracheal aspirates on postnatal day 1. A twofold increase was also observed in BPD lung tissue [10]. Mechanistic studies have shown that let-7b-5p, released by airway epithelial cells in response to hyperoxia, inhibits angiogenesis by suppressing its key target, *HMGA2* (High Mobility Group AT-Hook2), which is essential for mesenchymal cell proliferation and lung remodeling. Pharmacological inhibition of let-7 during hyperoxia exposure improved alveolar development in a neonatal mouse model, suggesting a causal functional link [10]. These preliminary findings suggest hsa-let-7b-5p as a candidate BPD biomarker and possible functional regulator in blood, tracheal aspirate, and lung tissue, pending peer-reviewed confirmation. The current study extends this evidence to a fourth biological matrix, saliva. At the target gene level, Zhang et al. identified *IGF1* as a direct target of let-7b-5p in pulmonary artery smooth muscle cells, demonstrating that let-7b-5p mimics suppress PDGF-induced proliferation and migration through *IGF1* downregulation [31]. IGF-1 and IGF-1R signaling are significantly reduced in BPD lungs and are required for the myofibroblast mechanosignaling that drives alveologenesis. Lee and Dutta demonstrated that let-7 family members repress *HMGA2* through six 3′-UTR binding sites, thereby reducing mesenchymal cell proliferation and lung remodeling capacity [32]. The development of BPD is primarily associated with hyperoxia in the underdeveloped lungs of premature infants [33]. Elevated levels of let-7b-5p induce several pathological changes, including inhibition of angiogenesis, disruption of alveolarization, and modulation of the TGF-β pathway, which contributes to fibrotic changes in the late stages of BPD [10]. Yang et al. profiled miRNAs across five stages of fetal rat lung development and explicitly linked developmentally regulated let-7 family members, including let-7b, to diseases of lung development such as BPD [34].

The let-7 miRNA family is widely recognized for its tumor suppressor function, with its levels frequently reduced in tumor tissues [35,36]. However, overexpression of hsa-let-7b-5p in the bloodstream has emerged as a marker of acute systemic pathologies. Notably, marked overexpression of this miRNA in serum exosomes strongly predicts the severity of coronary stenosis in patients with coronary artery disease, particularly under hyperglycaemic conditions [37]. Rapid increases in hsa-let-7b-5p levels have also been observed in acute pulmonary embolism as a response to endoplasmic reticulum stress [38]. Selective upregulation of plasma hsa-let-7b-5p has additionally been documented in a preclinical model of hemorrhagic stroke, where it was proposed as a candidate blood-based biomarker for distinguishing hemorrhagic from ischemic cerebrovascular injury [39]. Additionally, increased exosomal release of hsa-let-7b-5p has been documented in patients with immune thrombocytopenia, where it contributes to abnormal B-cell survival [40]. A 2025 study found that hsa-let-7b-5p levels are significantly higher in salivary exosomes from patients with binge-eating spectrum disorders than in healthy controls and that increased concentrations are directly associated with reduced oral microbial diversity and epigenetic modifications of the dopamine transporter gene *DAT1* [41]. Ambalavanan et al. reported upregulation of hsa-let-7b-5p in blood and tracheal aspirates, whereas Siddaiah et al. reported downregulation of the distinct family member hsa-let-7i-5p in tracheal aspirates in severe BPD [8]. This directional discordance highlights the tissue- and context-specific regulation of individual let-7 family members. This complexity must be explicitly acknowledged. Mechanistic studies by Ambalavanan et al. demonstrated that inhibition of let-7b-5p improves alveolar development, supporting a pathogenic rather than protective role for elevated let-7b-5p in BPD. This finding aligns with the upregulation observed in the BPD+ cohort [10].

Another member of the let-7 family, hsa-let-7c-5p, a key regulator of lung ontogenesis, cell proliferation, and adaptation to oxidative stress, was found to be overexpressed in infants’ saliva in this study. To date, no research has directly examined hsa-let-7c-5p expression in BPD or in the preterm lung in isolation. The plausibility of hsa-let-7c-5p as a candidate BPD biomarker is supported by the shared seed sequence and target gene repertoire of the let-7 family, its developmental lung expression profile, and its regulatory effects on TGF-β and RAS signaling. Yang et al. demonstrated that let-7 family members, including let-7c, are dynamically regulated during lung morphogenesis and are implicated in diseases of alveolar development [34]. Johnson et al. showed that the let-7 family, including let-7c, negatively regulates *RAS* oncogenes and noted that let-7c maps to chromosomal intervals deleted in lung cancers, positioning it as a relevant suppressor of uncontrolled cell growth in the pulmonary epithelium [36]. Wang et al. demonstrated that let-7c-5p targets components of TGF-β signaling in fibrotic disease [42]. TGF-β is a central driver of the fibrotic remodeling that characterizes late-stage BPD. While members of the let-7 family exhibit antiapoptotic effects under physiological conditions, excessive overexpression in the context of BPD may lead to the accumulation of dysfunctional cells and disrupt regenerative processes, resulting in abnormal tissue remodeling [8,34]. Recent studies indicate that, unlike in many other cancers, paradoxical overexpression of hsa-let-7c-5p occurs in oral squamous cell carcinoma, where it is secreted within exosomes into both saliva and the systemic circulation, promoting a malignant phenotype by suppressing the target protein TAGLN [43]. Furthermore, analysis of saliva from professional athletes demonstrated that hsa-let-7c-5p is included in a panel of miRNAs that are paradoxically and sharply overexpressed within the first 48 to 72 h following a concussion, serving as an early marker of neurotraumatic stress [44]. These findings reinforce the capacity of hsa-let-7c-5p to be overexpressed in biologically stressful conditions across diverse cellular compartments. *BMP4*, identified as a shared target in the miRNA–target interaction network (Figure 6), is a critical regulator of alveolar type II-to-type I transdifferentiation. Hyperoxia upregulates noggin to antagonize BMP4 signaling, thereby activating p53/p21-mediated senescence and contributing to alveolar arrest. The co-targeting of *BMP4* by let-7c-5p and other panel members represents a convergent mechanism consistent with impaired alveolarization in BPD.

In contrast to the let-7 family, hsa-miR-4454 expression in biological fluids is strongly associated with the development and metastasis of various malignant neoplasms. Oncoproteins from human papillomavirus directly induce overexpression of hsa-miR-4454, and elevated levels of this miRNA are associated with increased invasion and migration of cancer cells [45]. Elevated levels of hsa-miR-4454, originating from the endogenous retrovirus HERV-H, inhibit tumor suppressor genes and promote the growth of urinary tract tumors. As a result, hsa-miR-4454 and related miRNA panels have been proposed as biomarkers for carcinoma detection in contemporary machine learning-based serum diagnostic algorithms [46,47]. To date, hsa-miR-4454 has not been reported in any BPD-focused tracheal aspirate, blood, or lung tissue study. The detection of this marker in BPD+ saliva in the present study represents a preliminary observation specific to this biological matrix and requires independent replication before drawing definitive conclusions. In the salivary context, hsa-miR-4454 overexpression has been documented in the saliva of children with autism spectrum disorders, where elevated levels correlated with severe cognitive impairment and alterations in oral microbiome composition [48]. This cross-tissue relevance in saliva supports the biological plausibility of detecting this miRNA in the salivary matrix of preterm infants.

The convergence of all three identified miRNAs on molecular pathways independently established as relevant to BPD pathogenesis supports the biological plausibility of the miRNA panel. The validated target gene set, which includes 27,049 unique genes, encompasses IGF-1/IGF-1R signaling—suppressed in BPD lungs and targeted by let-7b-5p via *IGF1*—as well as *HMGA2*, which is essential for mesenchymal proliferation and lung remodeling and is repressed by the let-7 family. Additionally, TGF-β/BMP4 signaling, which drives alveolar arrest and fibrotic remodeling, is modulated by let-7c-5p, while the NF-κB inflammatory cascade, activated by hyperoxic injury, is modulated by miR-4454 through *SASH1* and the IL-17/NF-κB axis. Gene ontology enrichment analysis identified 15 significantly enriched biological process terms among the predicted targets, with the highest GeneRatio observed for the regulation of the DNA metabolic process. KEGG pathway enrichment analysis demonstrated significant enrichment in cell cycle regulation, ubiquitin-mediated proteolysis, autophagy, MAPK signaling, and focal adhesion pathways. While this pathway convergence does not constitute mechanistic proof of BPD causation in the current dataset, it establishes a biologically coherent framework that supports prioritization of experimental follow-up. Serum overexpression of hsa-let-7b-5p, hsa-miR-4454, and hsa-let-7c-5p is primarily associated with severe somatic conditions, including vascular disorders and malignant tumor progression. In contrast, salivary overexpression provides a distinct, non-invasive approach to assessing the local microenvironment and the central nervous system axis. The predicted miR-4454/NF-κB interaction is consistent with prior evidence that miR-4454 is clinically relevant to airway inflammation [49] and that NF-κB can regulate specific microRNA targets [50], together offering a plausible hypothesis for future experimental investigation. It does not, however, constitute evidence supporting the use of miR-4454 as a biomarker of alveolar damage severity or as a therapeutic target at this stage.

The identification of a candidate miRNA expression panel in saliva supports the emerging concept of a pathological lung–brain axis in bronchopulmonary dysplasia. Starke et al. demonstrated that hyperoxia-induced lung tissue injury and alveolar macrophage activation result in the secretion of extracellular vesicles containing ASC, which can cross the blood–brain barrier and transmit proinflammatory signals to the central nervous system [51]. Inter-organ miRNA transfer is mediated by extracellular vesicles. Hyperoxia-activated circulating extracellular vesicles have been shown to induce concurrent lung and brain injury in neonatal rat models [52]. This pathway has also been comprehensively reviewed in the context of preterm injury [53]. These findings suggest a biologically plausible route by which lung-derived miRNAs, including those identified in this study, could appear in saliva via systemic extracellular vesicle transport. The identified miRNAs, which regulate neurodegenerative processes, oxidative stress, and systemic inflammation, likely function as selective molecular components within these extracellular vesicles. The let-7 family appears to trigger neuroinflammation by modulating apoptotic and necroptotic signaling pathways, whereas miR-4454 may reflect the intensity of the systemic NF-κB-mediated response to tissue damage. Therefore, the salivary profile of these miRNAs may serve as a non-invasive indicator of local pulmonary pathology and elucidate mechanisms of distant interorgan interactions that contribute to the elevated risk of neurodevelopmental disorders in newborns with bronchopulmonary dysplasia. However, these observations remain speculative and require experimental confirmation.

Observed differences in birth weight and gestational age between the groups are consistent with global epidemiological data, confirming that extreme prematurity is the primary predictor of BPD. However, clinical heterogeneity among patients with ELBW or VLBW complicates the prediction of outcomes based solely on anthropometric measures. Principal component analysis showed that BPD+ samples formed a compact cluster along the first principal component, which accounted for 78.5% of the total variance, reflecting low within-group variability in miRNA expression. Control samples, in contrast, exhibited substantially greater dispersion, and complete separation between the groups was not achieved. This pattern is consistent with the possibility that salivary miRNA differences are already detectable before the formal clinical diagnosis of BPD at 36 weeks’ postmenstrual age. However, given the substantial intergroup differences in gestational age and clinical severity, this may equally reflect overall illness severity rather than a lung-specific process preceding injury. This study provides a focused foundation within a predominantly Central Asian, single-center cohort. Given the potential influence of genetic and regional variations, expanding this research to include independent cohorts from diverse geographic regions and ethnic groups is necessary to confirm the broader applicability of these preliminary results.

In summary, the overexpression of hsa-let-7b-5p, hsa-let-7c-5p, and hsa-miR-4454 in the saliva of low-birth-weight infants represents a candidate briomarker signal. The discriminatory performance of these miRNAs was internally validated by leave-one-out cross-validation. Because gestational age and birth weight are strongly linked to BPD status in this small cohort, potential confounding by prematurity could not be adequately evaluated, and the reported effect sizes—likely inflated by the small, significance-selected sample—should be regarded as exploratory pending external validation. The biological plausibility of all three candidates is grounded in established BPD-relevant target pathways. For hsa-let-7b-5p specifically, there is direct evidence of upregulation in three independent BPD biological matrices. These findings represent a hypothesis-generating signal for future risk stratification studies of BPD, rather than evidence of a ready-to-use early risk stratification tool. Accordingly, clinical translation requires external validation in larger, independent, multicenter cohorts with appropriate adjustment for confounding variables.

### Limitations of the Study

Several limitations inherent to the pilot design of this study must be considered when interpreting the findings. The cohort consisted of 20 infants, reflecting the strict inclusion criteria for the ELBW and VLBW population within a single-center context. Prospective validation in larger, multicenter cohorts is therefore necessary to confirm the generalizability of the identified panel and to facilitate the development of multivariable prognostic models. Relatedly, formal adjustment for clinical covariates was not performed due to sample size constraints. This represents a logical extension for future research. For the same reason, the ROC-derived performance estimates should be interpreted as preliminary benchmarks derived from the same discovery cohort used for miRNA selection and cutoff determination, which creates a risk of overfitting and optimism bias. As noted above, these estimates reflect diagnostic discrimination only. Calibration assessment, including a calibration plot and Brier score, was not performed and would be required before any calibrated risk prediction claim. External validation in independent cohorts is accordingly required before definitive diagnostic conclusions can be established. In a similar vein, as the study was conducted in a predominantly Central Asian population, further evaluation across diverse ethnic and clinical settings is essential to determine broader applicability. The design was sensitive only to very large effects (minimum detectable effect, Cohen’s d = 1.519 at 80% power) and was underpowered for small-to-moderate effects; observed effect sizes, estimated from the same small sample selected for significance, are likely overestimated and do not constitute evidence of adequate power. Furthermore, gestational age and birth weight, along with comorbidities such as necrotizing enterocolitis, disseminated intravascular coagulation, and posthaemorrhagic anemia, differed substantially between groups and are collinear with BPD status. Consequently, confounding by prematurity and its associated morbidities cannot be adequately evaluated in this cohort. The Spearman correlations reported here are descriptive and do not test confounding. Robust separation of a BPD-specific signal from prematurity will require a larger, gestational age- and weight-matched or propensity-adjusted study. A further limitation concerns the absence of PCR-based technical validation. The current findings are based solely on microarray discovery profiling, which offers broad transcriptome-wide coverage but is subject to cross-hybridization, probe-specific hybridization efficiency, and a comparatively narrower dynamic range than targeted quantitative approaches. Independent confirmation of the three candidate miRNAs by RT-qPCR in a new, non-overlapping sample set would substantially strengthen confidence in the reported differential expression estimates. This represents a necessary step before these candidates can be considered validated biomarkers, consistent with standard biomarker development pipelines that require orthogonal technical confirmation following microarray-based discovery. Separately, the use of passive, unstimulated saliva collection resulted in a relatively high sampling failure rate. Future studies should therefore evaluate standardized stimulation protocols that increase saliva yield while preserving microRNA integrity and minimizing potential effects on transcriptomic profiles. In addition, the higher prevalence of sepsis, NEC, and DIC in the BPD+ group underscores the need to include disease-specific comparator groups in future studies. Finally, the in silico pathway analyses provide a mechanistic framework that requires experimental confirmation through proteomic and functional approaches.

## 5. Conclusions

This pilot study identified a candidate salivary miRNA panel, comprising hsa-let-7b-5p, hsa-let-7c-5p, and hsa-miR-4454, whose expression was consistently elevated in extremely preterm infants who developed BPD. The minimally invasive nature of salivary sampling, combined with the biological plausibility of the identified candidates, supports the continued investigation of saliva as a discovery matrix for future BPD risk stratification research. This is further supported by their partial concordance with miRNA findings reported in tracheal aspirates and blood across the let-7 family.

As with any pilot investigation, the present findings should be interpreted within the boundaries of the study design. The modest sample size and single-center setting are inherent characteristics of this early-phase work, and prospective replication in larger, multicenter cohorts with covariate-adjusted analyses will be necessary to establish the generalizability of these results. Independent technical validation and external evaluation of candidate biomarker performance represent logical and well-defined next steps in the development of this biomarker panel.

Taken together, the results of this study provide a hypothesis-generating foundation for future research into minimally invasive molecular biomarkers of BPD. If replicated and externally validated in larger, independent cohorts, salivary miRNA profiling may eventually contribute to earlier and more individualized risk assessment in neonatal intensive care, though this remains a long-term goal rather than an immediate prospect.

## Figures and Tables

**Table 1:** General characteristics of the study group.

Variable	Overall *N* = 20	BPD+ *N* = 10	BPD− *N* = 10	*p*-Value
**Gestational age, weeks**	29.0 [27.0–31.5]	27.0 [26.0–28.0]	31.0 [29.0–32.0]	0.002
**Birth weight, g**	980.0 [692.5–1440.0]	692.5 [670.0–910.0]	1440.0 [990.0–1470.0]	0.001
**Gender**				0.350
female	13 (65%)	8 (80%)	5 (50%)	
male	7 (35%)	2 (20%)	5 (50%)	
**Apgar score at 1 min**	3.0 [2.0–4.5]	2.0 [2.0–3.0]	4.5 [3.0–5.0]	0.007
**Apgar score at 5 min**	5.0 [4.0–6.5]	4.0 [4.0–5.0]	6.5 [5.0–7.0]	0.004
**Silverman score at 1 min**	8.0 [7.0–8.0]	8.0 [7.0–8.0]	7.5 [7.0–8.0]	0.273
**Silverman score at 5 min**	8.0 [8.0–9.0]	8.0 [8.0–9.0]	8.0 [8.0–9.0]	0.436
**Intraventricular hemorrhage**				0.264
absence	4 (20%)	1 (10%)	3 (30%)	
presence	16 (80%)	9 (90%)	7 (70%)	
**Respiratory distress syndrome**				1.000
absence	2 (10%)	1 (10%)	1 (10%)	
presence	18 (90%)	9 (90%)	9 (90%)	
**Sepsis**				0.057
absence	13 (65%)	4 (40%)	9 (90%)	
presence	7 (35%)	6 (60%)	1 (10%)	
**Periventricular leukomalacia**				0.582
absence	16 (80%)	9 (90%)	7 (70%)	
presence	4 (20%)	1 (10%)	3 (30%)	
**Anemia of prematurity**				0.170
absence	12 (60%)	4 (40%)	8 (80%)	
presence	8 (40%)	6 (60%)	2 (20%)	
**Posthemorrhagic anemia**				0.033
absence	15 (75%)	5 (50%)	10 (100%)	
presence	5 (25%)	5 (50%)	0 (0%)	
**Necrotizing enterocolitis**				0.011
absence	14 (70%)	4 (40%)	10 (100%)	
presence	6 (30%)	6 (60%)	0 (0%)	
**Disseminated intravascular coagulation syndrome**				0.023
absence	10 (50%)	2 (20%)	8 (80%)	
presence	10 (50%)	8 (80%)	2 (20%)	
**Retinopathy**				0.011
absence	14 (70%)	4 (40%)	10 (100%)	
presence	6 (30%)	6 (60%)	0 (0%)	

Continuous variables: median [IQR]; categorical: *n* (%). Wilcoxon rank-sum test for continuous variables; Fisher’s exact test for categorical variables. Abbreviations: BPD+, infants with bronchopulmonary dysplasia; BPD−, control infants without BPD; IQR, interquartile range.

**Table 2:** Differential expression analysis of salivary miRNAs in preterm infants with bronchopulmonary dysplasia compared to controls.

miRNA Name	Species	log_2_FC	AveExpr	t	*p*-Value	adj. *p*-Value	B	Expression
hsa-let-7b-5p	Homo sapiens	4.45	7.44	5.49	<0.0001	0.001	3.99	Up-regulated in BPD
hsa-miR-4454	Homo sapiens	3.41	7.28	4.42	0.0001	0.011	1.21	Up-regulated in BPD
hsa-let-7c-5p	Homo sapiens	3.12	7.33	4.04	0.0003	0.021	0.24	Up-regulated in BPD

Abbreviations: log_2_FC, log_2_ fold change (BPD+ vs. BPD−); AveExpr, average log_2_ expression across all samples; t, moderated t-statistic; adj. *p*-value, Benjamini–Hochberg-adjusted *p*-value; B, log-odds of differential expression.

**Table 3:** Spearman rank correlation coefficients for associations between candidate miRNA expression levels, gestational age, and birth weight.

miRNA	Gestational Age		Birth Weight	
	r	*p*-Value	r	*p*-Value
hsa-let-7b-5p	−0.704	0.077	0.107	0.840
hsa-miR-4454	−0.111	0.812	0.536	0.236
hsa-let-7c-5p	−0.704	0.077	0.071	0.906

Spearman rank correlation coefficients (r) and corresponding two-tailed *p*-values are shown for each candidate miRNA in relation to gestational age and birth weight.

**Table 4:** Individual discriminatory performance of candidate salivary miRNAs evaluated by ROC curve analysis with internal validation.

miRNA	AUC (95% CI)	pAUC sp80	LOOCV AUC	DeLong *p*	Cutoff (log_2_RMA)	Se, %	Sp, %	PPV, %	NPV, %
hsa-let-7b-5p	0.937 (0.780–1.000)	0.824	0.944	<0.001	6.873	100	90.0	90.9	100
hsa-miR-4454	0.937 (0.770–1.000)	0.824	0.833	<0.001	8.185	100	90.0	90.9	100
hsa-let-7c-5p	0.905 (0.680–1.000)	0.735	0.833	<0.001	7.456	100	90.0	90.9	100

Abbreviations: AUC, area under the curve; CI, 95% confidence interval; pAUC sp80, partial AUC at ≥80% specificity; LOOCV, Leave-one-out cross-validation; Se, sensitivity (%); Sp, specificity (%); PPV, positive predictive value; NPV, negative predictive value.

**Table 5:** Statistical power analysis for candidate miRNAs.

miRNA	Cohen’s d	Effect Size
hsa-let-7b-5p	2.488	Very Large
hsa-miR-4454	2.060	Very Large
hsa-let-7c-5p	1.918	Very Large

Effect sizes are expressed as Cohen’s d and categorized according to standard benchmarks.

## Data Availability

The original data presented in the study are openly available in the GEO repository at https://www.ncbi.nlm.nih.gov/geo/query/acc.cgi?acc=GSE336455, accession GSE336455 (accessed on 29 June 2026).

## Supplementary Materials

The following supporting information can be downloaded at https://www.mdpi.com/article/10.3390/life16071202/s1, Table S1: Comprehensive data for all miRNAs identified in the study; Table S2: Contingency tables and classification count for candidate miRNAs at the optimal ROC cutoff; Table S3: List of target genes.

## Abbreviations

The following abbreviations are used in this manuscript:

AUC	Area Under the Curve
BPD	Bronchopulmonary Dysplasia
CI	Confidence Interval
DIC	Disseminated intravascular coagulation
ELBW	Extremely Low-Birth-Weight
FDR	False Discovery Rate
GEO	Gene Expression Omnibus
GO	Gene Ontology
HMGA2	High Mobility Group AT-Hook2
HERV-H	Human Endogenous Retrovirus subfamily H
HPV-16	Human Papillomavirus 16
IVH	Intraventricular hemorrhage
KEGG	Kyoto Encyclopedia of Genes and Genomes
log_2_FC	Log_2_ Fold Change
miRNA	Micro Ribonucleic Acid
NEC	Necrotizing Enterocolitis
NF-κB	Nuclear Factor Kappa B
NICU	Neonatal Intensive Care Unit
NPV	Negative Predictive Value
PCA	Principal Component Analysis
PPV	Positive Predictive Value
RMA	Robust Multi-array Average
ROC	Receiver Operating Characteristic
TGF-β	Transforming Growth Factor Beta
VLBW	Very Low-Birth-Weight
