# Supplementary material for: A Candidate Salivary miRNA Panel for Bronchopulmonary Dysplasia in Very and Extremely Low-Birth-Weight Preterm Infants: A Pilot Exploratory Study
Source: Life (Basel). 2026 Jul 21;16(7):1202. doi: 10.3390/life16071202 (PMC13413207; doi:10.3390/life16071202)
Supplement: Supplementary file 1 [file life-16-01202-s001.zip › Supplementary Table S2.pdf]

Supplementary Table S2. Contingency tables and classification count for candidate miRNAs at the optimal ROC cutoff.

**miRNA_Name**	**Threshold**	**TP**	**FN**	**FP**	**TN**	**BPD** (N)	**Control** (N)	**Se**	**Sp**
**hsa-let-7b-5p**	6.8726	10	0	1	9	10	10	1.000	0.900
**hsa-miR-4454**	8.1846	10	0	1	9	10	10	1.000	0.900
**hsa-let-7c-5p**	7.4561	10	0	1	9	10	10	1.000	0.900

Abbreviations: TP, true positive; FN, false negative; FP, false positive; TN, true negative; N, number of infants; Se, sensitivity; Sp, specificity.
